# A Potential Regulatory Role for Intronic microRNA-338-3p for Its Host Gene Encoding Apoptosis-Associated Tyrosine Kinase

**DOI:** 10.1371/journal.pone.0031022

**Published:** 2012-02-17

**Authors:** Aron Kos, Nikkie F. M. Olde Loohuis, Martha L. Wieczorek, Jeffrey C. Glennon, Gerard J. M. Martens, Sharon M. Kolk, Armaz Aschrafi

**Affiliations:** 1 Department of Cognitive Neuroscience, Donders Institute for Brain, Cognition and Behavior, Radboud University Nijmegen, Nijmegen, The Netherlands; 2 Department of Molecular Animal Physiology, Donders Institute for Brain, Cognition and Behavior, Radboud University Nijmegen, Nijmegen, The Netherlands; University of Florida, United States of America

## Abstract

MicroRNAs (miRNAs) are important gene regulators that are abundantly expressed in both the developing and adult mammalian brain. These non-coding gene transcripts are involved in post-transcriptional regulatory processes by binding to specific target mRNAs. Approximately one third of known miRNA genes are located within intronic regions of protein coding and non-coding regions, and previous studies have suggested a role for intronic miRNAs as negative feedback regulators of their host genes. In the present study, we monitored the dynamic gene expression changes of the intronic miR-338-3p and miR-338-5p and their host gene Apoptosis-associated Tyrosine Kinase (AATK) during the maturation of rat hippocampal neurons. This revealed an uncorrelated expression pattern of mature miR-338 strands with their host gene. Sequence analysis of the 3′ untranslated region (UTR) of rat AATK mRNA revealed the presence of two putative binding sites for miR-338-3p. Thus, miR-338-3p may have the capacity to modulate AATK mRNA levels in neurons. Transfection of miR-338-3p mimics into rat B35 neuroblastoma cells resulted in a significant decrease of AATK mRNA levels, while the transfection of synthetic miR-338-5p mimics did not alter AATK levels. Our results point to a possible molecular mechanism by which miR-338-3p participates in the regulation of its host gene by modulating the levels of AATK mRNA, a kinase which plays a role during differentiation, apoptosis and possibly in neuronal degeneration.

## Introduction

MicroRNAs (miRNAs) constitute a novel class of small 21–23 nucleotides long, non-coding RNAs that act as post-transcriptional regulators of gene expression. They are highly conserved during evolution, and involved in a wide variety of biological processes. For example in developmental processes, apoptosis, metabolism, cell differentiation, and morphogenesis [1], [2], [3], [4]. In animals, miRNAs regulate gene expression by base pairing imperfectly to the 3′ untranslated region (UTR) of target mRNAs, thereby inhibiting protein synthesis or causing mRNA degradation [5].

Although most miRNAs are encoded in intergenic regions or within exonic loci, approximately one-third of the mammalian miRNA genes are located in introns of non-coding RNA genes, or within introns of protein-coding genes [6]. They are referred to as intronic or intragenic miRNAs [7]. While the majority of the mammalian intronic miRNAs are transcriptionally linked to their host gene expression and are processed from the same primary transcript, computational surveys suggested that one fourth of intronic miRNAs are transcribed from their own promoters [8]. The precursor miR-338 sequence is intronically encoded within the Apoptosis-associated Tyrosine Kinase (AATK, also known as AATYK) host gene [9]. This gene is upregulated during apoptosis of myeloid precursor cells induced by interleukin-3 deprivation [10], [11], and in cultured cerebellar granule neurons undergoing apoptosis induced by exposure to a low K^+^ environment [12].

**Figure 1:**
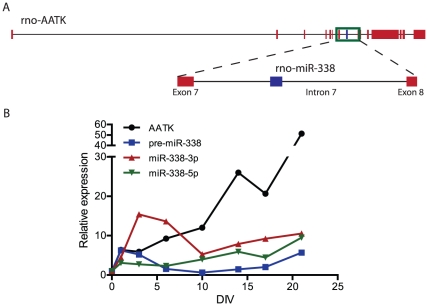
MiR-338 is encoded within the AATK gene and is expressed during maturation of hippocampal neurons. (A) A schematic overview of rat miR-338 encoded within the seventh intron (depicted in blue) of the AATK gene located on chromosome 11, with the exons shown in red. The depicted genes are *Rattus norvegicus* AATK (rno-AATK) and miR-338 (rno-miR-338). (B) qPCR assay was used to assess levels of pre-miR-338, mature miR-338-3p and miR-338-5p, and AATK mRNA in cultured rat hippocampal neurons (DIV 0–21). The data represents relative fold change in AATK and miR-338 expression levels to DIV 0.

Transcription, splicing and further processing will produce mature miR-338-3p and miR-338-5p from the seventh intron of the AATK gene (Figure 1A). For most miRNAs, only one strand (the guide strand) of the double-stranded miRNA duplex is loaded into RISC, while the other (*) strand is destroyed rapidly [13]. However, in some cases such as for miR-338, both strands (5p and 3p) are selected, and can function as post-transriptional repressors [14]. Both AATK and miR-338 are highly conserved genes, and prominently expressed within the vertebrate central nervous system (CNS) [12], [15]. Little is known about the role of miR-338 in maintaining neuronal function. Recent studies have indicated a role for miR-338-3p in oligodendrocyte differentiation and maturation [16]. In addition, miR-338-3p is enriched in distal axons, where it modulates mitochondrial function, and consequently oxygen dependent metabolic pathways in sympathetic neurons by regulating the expression levels of cytochrome *c* oxidase, subunit IV [17], [18].

Since previous studies have also demonstrated a role for AATK in stimulating neuronal differentiation [19], we here monitored the gene expression changes of precursor (pre-) and mature miR-338 strands and their host gene (AATK) during the first 21 days *in vitro* (DIV) neuronal differentiation. This investigation revealed an uncorrelated expression pattern of the intronic miR-338-3p, and -5p with their host gene. Follow-up bioinformatic surveys identified that the 3′UTR of rat AATK mRNA contains two putative binding sites for miR-338-3p, suggesting that this miRNA may regulate the expression of its host gene during neuronal differentiation or degeneration. In the current study, gene expression analysis was combined with luciferase-based gene activity assay, to further examine the functional association of miR-338-3p and miR-338-5p in relation to their host gene.

## Results

### Profiling miR-338 and AATK Expression in Hippocampal Neurons during Differentiation in vitro

Previous reports have demonstrated that retinoic acid-mediated neuronal differentiation of human neuroblastoma cells results in the synchronized induction of expression levels of miR-338-3p and its host gene AATK [9]. Although both miR-338 and AATK are known to be specifically expressed in neuronal tissue [15], [19], [20], little is known about their relative abundance during neuronal maturation and neurite outgrowth. To examine whether the onset of miR-338 expression in hippocampal neurons in culture was correlated with the expression of AATK, the expression levels of the pre- and mature miR-338 strands, as well as AATK during *in vitro* differentiation of these neurons were investigated. A comparative qRT-PCR experiment was performed on dissociated embryonic day 18 (E18) hippocampal neurons at eight different maturational stages (Days *in vitro* (DIV) 0, 1, 3, 6, 10, 14, 18, and 21; Figure 1B). In rat hippocampal neurons, the expression levels for pre-, and mature miR-338 strands remained at significantly lower levels as compared to AATK mRNA levels. While the levels of miR-338-5p, continuously elevated within the assessment period (ten-fold until DIV 21), miR-338-3p levels increased only during early neuronal differentiation (fifteen-fold until DIV 6). Afterwards until DIV10, miR-338-3p expression levels decreased slightly, and remained at a relatively low level throughout the differentiation period (measured up until DIV 21). In addition, pre-miR-338 levels increased slightly within the first day in culture, followed by gradually decreased levels between DIV 3 and DIV 14, when pre-miR levels resumed to DIV 1 levels. Conversely, the relative levels of AATK mRNA increased considerably during the onset of neuronal differentiation (up to DIV 6), and AATK expression levels increased approximately sixty-fold within the first 21 days of *in vitro* differentiation (Figure 1B). The outcome of this experiment strongly suggests that the AATK mRNA levels and the levels of the (pre, -3p, -5p) miR-338 in rat hippocampal neurons are not coordinately regulated.

### AATK Is a Target of miR-338 in Neurons

**Figure 2:**
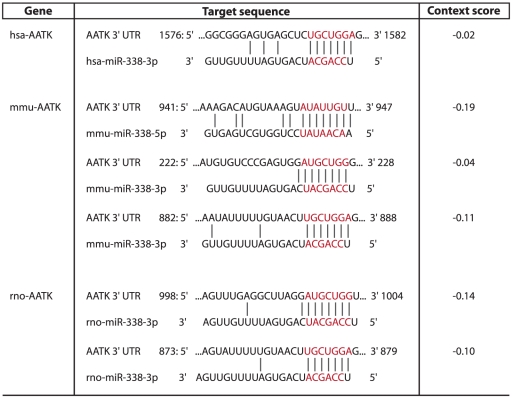
An overview of *in silico* identified putative miR-338 target sites within the AATK 3′UTR. Three AATK genes are depicted namely *Homo sapiens* AATK (hsa-AATK), *Mus musculus* AATK (mmu-AATK) and *Rattus norvegicus* AATK (rno-AATK). The miR-338 seed sequence is indicated in red.

To evaluate whether the expression of miR-338 and AATK mRNA is functionally related, the possibility that miR-338 has the capacity to regulate AATK mRNA expression was considered. To initially explore this postulate, the TargetScan algorithm [21], [22] was used to search for miR-338 binding sites in the 3′UTR of AATK mRNA. This *in silicio* analysis identified two 7-mer binding sequences within the 3′UTR of rat AATK mRNA which have the potential to function as a putative binding site for miR-338-3p (Figure 2). Furthermore, the 3′UTR of mouse AATK mRNA was found to contain two putative *cis*-acting binding sites for miR-338-3p, and one putative binding site for miR-338-5p. Moreover, the 3′UTR of human AATK mRNA contained a conserved sequence complementary to the seed target region of miR-338-3p (Figure 2).

**Figure 3:**
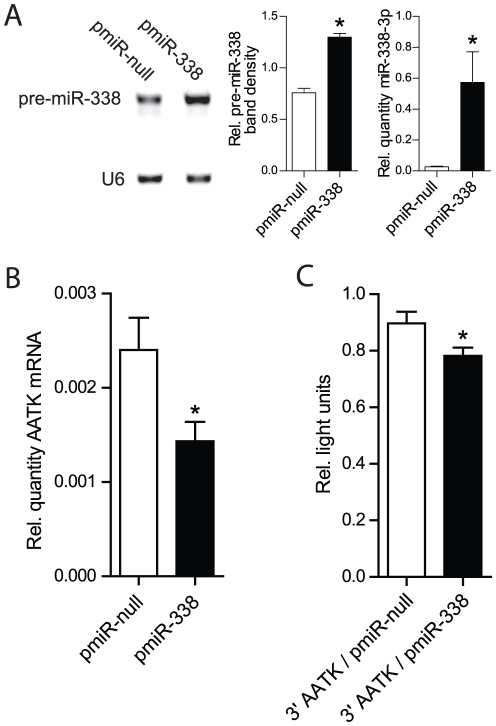
MiR-338 targets AATK. (A) Pre-miR-338, and miR-338-3p levels in B35 cells (transfected with pmiR-338 or pmiR-null plasmids) were quantified following PCR. Pre-miR-338 levels are visualized on 4% agarose gels containing ethidium bromide using UV absorption (254 nm wavelength), and pre-miR-338 band intensities are expressed relative to U6 snRNA. Furthermore qRT-PCR assessment of miR-338-3p levels, expressed relative to U6 snRNA following pmiR-338 overexression versus the null condition. (B) Quantification of AATK mRNA levels in B35 cells transfected with pmiR-338 or pmiR-null vectors, as determined 72 hrs following transfection using qRT-PCR. (C) Relative firefly luciferase activity in B35 cells measured in light units. Cells were co-transfected with luciferase encoding the 3′UTR of rat AATK (indicated as 3′AATK) and either with the pmiR-null control vector, or the pmiR-338 overexpression vector. Luciferase activity was normalized to Renilla luciferase activity. Error bars represent the SEM for n = 3 independent experiments, * is *p*<0.05 with pmiR-null vs. pmiR-338 (Student's t test).

To explore whether miR-338 regulates AATK mRNA levels in neurons, AATK mRNA levels were monitored after transfecting rat B35 neuroblastoma cells with a miR-338 expression vector. MiR-338 transfection resulted in a significant increase in the levels of pre-miR-338, and mature miR-338-3p levels as compared with the endogenous miR-338 levels in null vector-transfected neuroblastoma cells (Figure 3A). In miR-338 overexpressing cells, AATK mRNA levels decreased by 30% when compared with null-vector-transfected neurons (Figure 3B). To assess whether miR-338 can specifically target AATK mRNAs, B35 cells were co-transfected with the miR-338 expression vector and a luciferase reporter plasmid containing the rat AATK 3′UTR. The presence of the miR-338 expression vector significantly reduced luciferase activity by 15% in B35 cells as compared to null-vector co-transfected neurons, indicating that 3′UTR of AATK mRNA is targeted by miR-338 (Figure 3C).

**Figure 4:**
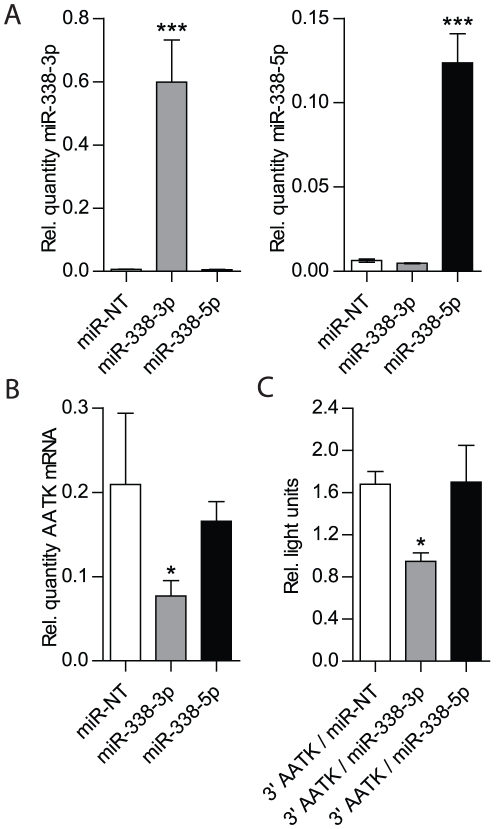
Regulation of AATK by mature miR-338 Strands. (A) Relative mature miR-338 levels in B35 cells transfected with either miR-338-3p, or miR-338-5p mimics, as compared to endogenous miR-338 (-3p, and -5p) levels in miR-NT transfected control cells. (B) AATK mRNA levels in B35 cells following transfection with mature miR-338 mimics (-3p, or 5p), as compared to host gene levels in miR-NT transfected B35 cells. Mature miR-338 levels are expressed relative to U6 snRNA, whereas AATK levels are normalized to the levels of β-actin. Error bars represent the SEM for n = 3 independent experiments. (C) Relative firefly luciferase activity in B35 cells measured in light units. B35 Cells were co-transfected with luciferase encoding the 3′UTR of rat AATK (indicated as 3′AATK) and either miR-338-3p, miR-338-5p mimics, or with miR-NT serving as non-targeting control ribo-oligonucleotides. Error bars represent the SEM for n = 8, * is *p*<0.05 and *** is *p*<0.0001 (one-way ANOVA with Bonferroni multiple comparison test).

The initial results derived from B35 cells transfected with the miR-338 vector suggest that miR-338 has the capacity to modulate AATK mRNA levels. To specifically delineate the contribution of mature miR-338-3p, or miR-338-5p in reducing AATK mRNA levels, we individually lipofected double-stranded miR-338-3p and miR-338-5p mimics into B35 cells. Transfection of miR-338-3p and -5p resulted in an approximately hundredfold increase in mature miR-338 levels, as compared to the endogenous miR-338 levels in non-target miRNA (miR-NT)-transfected neuroblastoma cells (Figure 4A). As shown in Figure 4B, in miR-338-3p transfected cells a significant reduction of AATK mRNA levels was achieved. Conversely, overexpression of miR-338-5p did not alter AATK levels significantly, as compared to the AATK mRNA levels of miR-NT transfected control samples. To further substantiate this finding, we co-transfected B35 cells with the luciferase reporter plasmid containing the AATK 3′UTR combined with either miR-338-3p, or with miR-338-5p. When compared to the control conditions, the introduction of the miR-338-3p reduced luciferase activity ∼50% (Figure 4C). In contrast, luciferase levels did not change significantly when the miR-338-5p mimic was co-transfected with this reporter plasmid, indicating that rat AATK mRNA is specifically targeted by miR-338-3p. The outcome of these studies indicate that while overexpression of miR-338-5p may have modest, although not significant, effects on AATK mRNA levels, most pronounced reduction of the host gene mRNA levels is observed following the overexpression of miR-338-3p in B35 cells.

## Discussion

**Figure 5:**
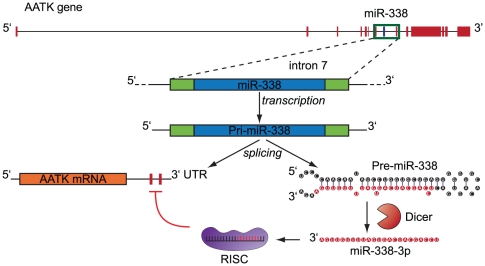
Proposed model of AATK regulation by its intronic miR-338. MiR-338 encoded within the seventh intron of the AATK gene is transcribed from the genome (blue block). Subsequent splicing generates the miR-338 precursor hairpin followed by Dicer-mediated maturation, leading to the incorporation of the mature miR-338-3p strand into the RISC complex. This ribonucleoprotein complex targets *cis*-acting binding sites for miR-338-3p (red blocks) located on the 3′UTR of AATK mRNA, resulting in the degradation of the transcript (orange block).

The outcome of this study puts forward the idea that an intronic miRNA may have the capacity to regulate the expression of its host gene. In agreement with previous measurements, we find that the average level of AATK repression is modest. Interestingly, van Oudenaarden and associates recently demonstrated that regulation by miRNAs establishes a threshold level of target mRNA below which protein production is highly repressed. Near this threshold, protein expression responds sensitively to target mRNA input, consistent with a mathematical model of molecular titration, suggesting that miRNAs can act both as a switch and as a fine-tuner of gene expression [23]. Previous studies revealed an increased expression of AATK mRNA and protein during postnatal brain development, and elevated levels of AATK have been demonstrated to enhance neurite outgrowth [24]. Interestingly, it has been shown that AATK up-regulation is also associated with cultured apoptotic cerebellar granule neurons [11]. These findings suggest that specific amounts of AATK may be important for proper neuronal growth and homeostasis. A recent paper has suggested that miR-338 is involved in the control of neuroblast apoptosis and in neuroblastoma pathogenesis [25]. Thus, miR-338 mediated fine-tuning of AATK expression levels during the onset of neuronal differentiation and apoptosis may be an important physiological mechanism to control differentiation and the number of neurons. Previous studies have suggested that approximately 20% of intragenic miRNAs have the capacity to target their host mRNA transcript [26]. Further, Kyoto Encyclopedia of Genes and Genomes (KEGG) pathway analysis revealed that 22 out of 74 pathways implicated the association of host genes, demonstrated significant over-representation of proteins encoded by the mRNA targets of associated intragenic miRNAs [7]. Similar to many intronic miRNAs, miR-338 lacks its own promoter and is therefore processed out of its intronic sequence [27], [28]. Previous investigations have revealed that the proportion of intronic miRNAs whose expression profiles are synchronized with their host genes ranges between 34%–71% [29], [30]. Here we propose a model, in which one of the two complementary versions of mature miR-338, namely miR-338-3p, generated through splicing and Dicer-mediated maturation, has the capacity to modulate the expression level of its host gene AATK in rat neuroblastoma cell lines (Figure 5). This outcome is in agreement with bioinformatics analyses shown in Figure 2, in which the miR-338-5p binding site is restricted to the 3′UTR of the mouse homologue of AATK mRNA, and is very poorly conserved evolutionary. For example, the -5p binding site is absent in the 3′UTRs of rat and human versions of AATK mRNA. Despite the lack of a *cis*-acting binding site for miR-338-5p in rat AATK mRNA, our studies indicate that overexpression of this mature miRNA resulted in a modest reduction of host gene mRNA levels in rat neuroblastoma cell lines. While the exact mechanism for this observation is not clear, we presume that reduced AATK levels could be explained by secondary effects inherent with the overexpression of this mature miRNA. This notion is further supported by our observation that luciferase activity upon miR-338-5p introduction remained unchanged, suggesting that miR-338-5p lacks the capacity to directly modulate AATK levels through interacting with its 3′UTR. A detailed survey of miR-338-5p targets using the *in silico* TargetScan tool reveals that this miRNA has number of transcription regulators (such as SP3, and SP2 transcription factors) as putative targets, which could be modulated in their expression and subsequent function upon miR-338-5p overexpression, resulting in altered AATK mRNA transcription.

Recent studies have suggested a potential regulatory role of co-expressed intronic miRNAs with their host gene. Furthermore, a few studies have suggested a functional relationship between miRNA host genes and putative targets of corresponding intronic miRNAs. For example, the heart-specific host gene Myh6 is co-expressed with the intronic miR-208a, the latter of which has the capacity to regulate thyroid hormone associated protein 1 and myostatin, both negative regulators of muscle growth and hypertrophy [31]. Furthermore, the intron of the schizophrenia-susceptibility gene GRID1 encodes miR-346 which is down-regulated in schizophrenia, and based on target prediction algorithms preferentially targets genes which may be involved in the pathophysiology of this disorder [32].

In conclusion, the current investigations have determined the expression pattern of miR-338 and its host gene AATK during *in vitro* differentiation of primary hippocampal neurons and assessed the possible regulation of AATK by miR-338-3p. Collectively, these results suggest that miR-338-3p has the capacity to modulate rat AATK mRNA levels. MiR-338-3p-dependent regulation of AATK mRNA would thus offer a mechanism to control availability of this neuronal mRNA during neuronal differentiation and degeneration. This conjecture is presented here, as a testable hypothesis that we suggest should be subject to future experimental examination.

## Materials and Methods

### Bioinformatic analysis

The Targetscan algorithm [33] was used to interrogate the 3′UTR sequence of AATK mRNA for putative binding sites of miR-338. The context scores indicated in Figure 2 were calculated by the TargetScan algorithm [34]. In short, the context score combines the 3′ pairing score, local AU content and distance from the nearest 3′UTR terminus to provide an interaction prediction in which a lower context score indicates a higher targeting preference by a miRNA.

### Transfection of DNA constructs and miR-338 mimics

The miRNASelect pEGP-mmu-mir-338 and its corresponding negative control vector pEFP-mir-null expression vectors were commercially obtained from Cell Biolabs (San Diego). The 3′UTR from the AATK gene was amplified from a rat cDNA library using the following primers incorporating the SacI and Xbal restriction sites: AATK 3′UTR forward, AAAAAAAAGAGCTCTGAGACCCAGGTTATCCCAC; AATK 3′UTR reverse, AAAAAAAATCTAGAGGAACAAGAAAATCATTGCA. The AATK 3′UTR amplicon was ligated into the pmirGLO Dual-Luciferase miRNA target expression vector (promega) between the SacI and XbaI restriction sites. Transfection of DNA constructs into cell lines was performed using Lipofectamin 2000 reagent (Life Technologies) according to the manufacturer's instructions. The double-stranded RNA that mimics endogenous rat miR-338-3p [UCCAGCAUCAGUGAUUUUGUUGA], rat miR-338-5p [AACAAUAUCCUGGUGCUG AGUG], and miR-NT, used as a non-targeting control, were obtained from Qiagen. The introduction of miRNA mimics was accomplished by lipofection using siPORT NeoFX (Life Technologies), with a 30 nM miRNA mimic concentration per condition.

### Reverse transcription and Real-Time PCR

Total RNA was isolated using TRIzol (Life Technologies), according to the protocol provided by the manufacturer. The purity of all isolated RNA samples was determined by agarose gel electrophoresis and UV-spectrophotometric analysis, respectively. The mean ± S.D. of the 260/280 nm ratios was 2.0±0.05. Contamination by genomic DNA was removed by treating 1 µg of each RNA sample with 2 U deoxyribonuclease (DNase) (Sigma Aldrich, D7691) for 1 hr at 37°C, followed by DNase inactivation at 65°C for 10 min. cDNA was synthesized from 0.5–1 µg RNA according to the protocol provided with the revertAid First Strand cDNA Synthesis Kit (Fermentas). For detection of mature miR-338 (-3p, and -5p), the miScript reverse transcription kit (Qiagen) was utilized. Real-time PCR was performed with 1/10 diluted cDNA using the Maxima SYBR Green/ROX qPCR master mix (Fermentas) or the miScript SYBR green PCR kit (Qiagen) for detection of mature miR-338 (-3p, and -5p). The following gene-specific primers were used: AATK forward, ATGCTGGCCTGCCTGTGTTGT; AATK reverse, AGGGGCAGGACATACACATCGG; pre-miR-338 forward, AACAATATCC TGGTGCTGAGTG; pre-miR-338 reverse, CAACAAAATCACTGATGCTGGA; mature miR-338-3p forward, TCCAGCATCAGTGATTTTGTTG; mature miR-338-5p forward AACAATATCCTGGTGCTG AGTG; β-Actin forward, CCAGATCATGTTTGAGACCTTC; β-Actin reverse, AGGATCTTCATGAGGTAGTCTG; U6 forward, GCTTCGGCAGCA CATATA; U6 reverse, CGCTTCACGAATTTGCGT. Relative gene expression differences were calculated by applying the delta C_T_ method [35]. DNA band intensities on an agarose gel were quantified using LabWorks image acquisition software provided with an EpiChemi II Darkroom gel documentation system (UVP). Equal regions of interest were selected to obtain the band intensities after background substraction. U6 band intensities were used to normalize pre-miR-338 measurements.

### Luciferase assay

Three days after transfection, the cells were lysed and processed for luciferase luminescence measurements. For detection of luciferase activity the Dual-Glo luciferase assay system (Promega) was performed as previously described [36]. Briefly, an appropriate amount of Dual-Glo reagent was added to the cell medium enabling cell lysis and subsequent detection of firefly luminescence in a luminometer. Normalization of the samples was performed by addition of the Dual-Glo Stop & Glo reagent enabling the detection of renilla luminescence, and the luciferase activity in relative light units (RLU) was subsequently calculated.

### Cell culture

Rat neuroblastoma B35 cells (rat CNS derived) were obtained from American Type Culture Collection (Manassas, VA, USA), and were cultured in Dulbecco's Modified Eagle Medium (DMEM) high glucose (4.5 g/L)supplemented with pyruvate (10 mg/mL), penicillin/streptomycin antibiotics (20 µg/mL) and 10% fetal calf serum. The cells were maintained at 37°C and 5% CO_2_. Primary cultures of hippocampal neurons were prepared from embryonic day 18 rats as described [37], and maintained in a neurobasal medium supplemented with B27 (Invitrogen, Carlsbad, CA, USA) and 2 mmol/L GlutaMax (Life Technologies).

### Statistics

Quantitative data are presented as the mean ± SEM. Student's *t* test was used to determine significant differences between two groups. One-way ANOVA with Bonferroni's multiple comparison test was used to analyze significant differences among multiple groups; *p*≤0.05 was considered significant.
